# Associations of Hormonal and Metabolic Parameters with Bone Mineralization in Young Adult Females

**DOI:** 10.3390/nu15112482

**Published:** 2023-05-26

**Authors:** Martyna Patalong-Wójcik, Anna Golara, Alicja Sokołowska, Katarzyna Zając, Mateusz Kozłowski, Mariola Krzyścin, Agnieszka Brodowska, Igor Syrenicz, Aneta Cymbaluk-Płoska, Elżbieta Sowińska-Przepiera

**Affiliations:** 1 Department of Endocrinology, Metabolic and Internal Diseases, Pomeranian Medical University in Szczecin, Unii Lubelskiej 1, 71-252 Szczecin, Poland; patalongmartyna@gmail.com (M.P.-W.); krzyscin@tlen.pl (M.K.); igor.syrenicz@gmail.com (I.S.); elzbieta.sowinska.przepiera@pum.edu.pl (E.S.-P.); 2 Department of Reconstructive Surgery and Gynecological Oncology, Pomeranian Medical University in Szczecin, Al. Powstańców Wielkopolskich 72, 70-111 Szczecin, Poland; alasok99@gmail.com (A.S.); kasiazajac24@onet.pl (K.Z.); mtkoozo@gmail.com (M.K.); aneta.cymabluk@gmail.com (A.C.-P.); 3 Department of Gynecology, Endocrinology and Gynecological Oncology, Pomeranian Medical University in Szczecin, Unii Lubelskiej 1, 71-252 Szczecin, Poland; agabrod@wp.pl; 4 Pediatric, Adolescent Gynecology Clinic, Department of Gynecology, Endocrinology and Gynecological Oncology, Pomeranian Medical University in Szczecin, Unii Lubelskiej 1, 71-252 Szczecin, Poland

**Keywords:** hormonal, metabolic parameters, estradiol, cortisol, bone mineralization, osteoporosis, sclerostin

## Abstract

Osteoporosis is characterized by impaired bone mineralization and microarchitecture. An important protective factor is a high peak bone mass (PBM), attained in the second and third decade of life. The aim of the study was to evaluate the effect of hormonal and metabolic parameters on bone mineralization in young adult female patients. A total of 111 participants qualified for the study. Bone mineral density of the lumbar spine (L1–L4) and whole skeleton was measured using dual-energy X-ray absorptiometry (DXA). Hormonal parameters were determined: the concentrations of androstendione, dihydroepiandrosterone sulphate, testosterone, sex hormone binding protein, 17-OH-progesterone, folliculotropic hormone, estradiol, thyrotropic hormone, free thyroxine and cortisol. Metabolic parameters were also examined. The study showed a statistically significant correlation between bone mineral density and estradiol concentration and a negative relationship between cortisol concentration and the bone mineral density (BMD) Z-score of the lumbar spine. Sclerostin measurements taken during this study were not related to bone mineral density. It has been shown that the concentration of the hormones tested, even within the reference range, may affect bone mineralization. We suggest observing the follow-up of the menstrual cycles, as well as analyzing the results of test patients in an annual examination system. However, each clinical case should be considered individually. The sclerostin test is currently not useful in the clinical evaluation of bone mineralization in young adult women.

## 1. Introduction

Sex hormones are a key factor in normal skeletal development; however, the underlying mechanisms remain incompletely understood [1,2]. These steroids interact with specific receptors: the androgen receptor (AR) and the estrogen receptor (ER), which are present on the surface of all types of bone cells. Some of the actions induced by these receptors are ligand-independent, especially the stress response [3].

Estrogens (E) are the main regulators of bone mass in both men and women. They are responsible for preserving bone mass, balancing remodeling processes, reducing bone turnover, responding appropriately to mechanical loading and promoting the bone-forming cell function while inhibiting osteogenic cells [4]. A direct correlation between low estradiol values and low bone mineralization has also been shown in premenopausal women [5]. Sex hormone receptor polymorphisms most likely determine the sensitivity and response of target tissues. Among others, a BMD dependence on the ER-alpha variant and an increased risk of osteoporosis dependent on the ER- beta polymorphism have been observed [4]. Important target cells for sex steroids are osteocytes. It is believed that estrogens acting on ER reduce sclerostin production [6]. Estrogen treatment of postmenopausal women resulted in a decrease in sclerostin [7]. Estrogen deficiency quadrupled the apoptosis of osteocytes, which in the process released accumulated factors (including RANKL), enhancing remodeling and resorption [3,8]. Under conditions of estrogen deficiency, an increase in osteoblast apoptosis, increased oxidative stress and decreased bone matrix formation was observed [8]. In addition, E stimulates osteoprotegerin production by increasing its mRNA concentration, leading to inhibition of resorption. Such effects are not shown by androgens [1,4,7]. Studies on the effect of muscle on the skeleton confirm a linear, direct proportional relationship between bone mineral density and lean body mass. This effect is greater in women between puberty and menopause, which is explained by the influence of sex hormones. After menopause, the accumulated bone gives way to the remodeling processes. The action of E also occurs through a stimulatory interaction with the growth hormone (GH)/ insulin-like growth factor (IGF-1) axis [2,9]. In addition, E directly stimulate IGF-1 production in the liver, an effect that androgens do not have [3].

Androgens do not affect osteocyte function significantly [10], but directly stimulate osteoblasts [10,11]. They increase their proliferation and differentiation, part of this effect being mediated through IGF-1 [4]. Findings on the effect of androgens on mechanotransduction are contradictory [3,12]. Androgens have a positive effect on the musculoskeletal system, which stimulates the development and maintenance of adequate skeletal properties [3]; however, this relationship mainly applies to men [13]. A significant positive correlation has been shown between dihydroepiandrosterone sulphate (DHEA-S) concentrations and markers of bone turnover (both resorption and formation) in both sexes, especially before peak bone mass is achieved. Its mechanisms of action are complex, and most likely include the stimulation of androgen receptors, enhancement of IGF-1 secretion and inhibition of pro-resorptive IL-6 [12].

Sclerostin is a protein secreted by the most numerous and active bone cells: the osteocytes. It is an inhibitor of the canonical Wnt-beta catenin pathway, thereby inhibiting bone formation. This action on osteoclasts reduces osteoprotegerin secretion giving consent to osteoclast activation The anti-sclerostin antibody, romosozumab, was approved by the European Medicines Agency (EMA) in 2019 for the treatment of osteoporosis [14]. Given this, sclerostin appears to be a promising marker for assessing bone mass. However, the vast majority of studies assess sclerostin concentrations in older adults, nursing home residents or, conversely, young athletes or patients with primary skeletal diseases. The data on young adults is scarce.

In the following study, we present the correlation between hormonal and metabolic parameters and bone mineralization. We also investigated the correlation between sclerostin concentration and hormonal parameters, and assessed whether sclerostin could be useful in clinical practice as an exponent of bone mineralization.

## 2. Materials and Methods

### 2.1. Participation in the Study

A total of 130 women who sought counseling for transient menstrual disorders at the Gynaecological Endocrinology Outpatient Clinic of the Department of Endocrinology, Metabolic Diseases and Internal Medicine at the Pomeranian Medical University in Szczecin, for whom organic causes of the reported abnormalities were excluded, were initially enrolled in the study. Additionally, 103 female volunteers without menstrual cycle issues were part of the program. Aged 20 to 30 years, Caucasian woman, a history of normal puberty, not using a daily medication, no obvious physical anomalies, and patient agreement were the inclusion criteria. Exclusion criteria included endocrine diseases that affect bone mineralization, such as thyroid disorders and premature ovarian function suppression (POF), severe systemic diseases that affect bone mineralization, such as rheumatological diseases (RA), severe lung diseases, gastrointestinal disorders (including coeliac disease), diabetes mellitus, kidney diseases, etc., past or present eating disorder, and genetic or metabolic conditions linked to bone mineralization disorders. A total of 111 female participants were ultimately approved for the study and the analysis, based on the aforementioned requirements.

The Pomeranian Medical University in Szczecin’s Bioethics Committee gave its clearance for the study (Resolution KB-012/78/18 of 18.06.2018).

The data was collected between June 2018 and June 2021.

### 2.2. Basic Procedures

1. A standard subject and physical examination was performed on all patients. Body mass index (BMI (kg/m^2^)) was calculated using anthropometric measurements (height (cm) and weight (kg)).
2. Hormonal parameters were determined from fasting venous blood collected from the basilic vein in the morning between the 2nd and the 6th day of the follicular phase of the menstrual cycle: concentrations of androstendione, dihydroepiandrosterone sulphate (DHEA-S), testosterone (T), sex hormone binding protein (SHBG), 17-OH-progesterone, folliculotropic hormone (FSH), estradiol (E), thyrotropic hormone (TSH), free thyroxine (fT4) and cortisol. Based on the results, the free androgen index (FAI), free testosterone (FT) and bioavailable testosterone (BT) concentrations were calculated.

Variables of androgen parameters: free testosterone (FT) and bioavailable testosterone (BT) were calculated, using an online calculator: http://www.issam.ch/freetesto.htm (accessed on 10 April 2023). Free androgen index (FAI) was calculated, using the formula:

$$FAI=\frac{T[ng/mL]\times 347}{SHBG\;nmol/l}$$

In addition, metabolic parameters were assessed: fasting glucose and insulin levels, total cholesterol (CHOL), LDL and HDL cholesterol and triglycerides (TG). The above-mentioned tests were performed in a hospital laboratory using standard methods: electrochemiluminescence (ECLIA) Roche test used in the Cobas device, immunoenzymatic (ELISA), chemiluminescence-immunoassay (CLIA) performed on the LIAISON XL device and calorimetric methods. The HOMA-IR insulin resistance index was calculated using the HOMA2 Calculator software (https://www.rdm.ox.ac.uk/about/our-clinical-facilities-and-mrc-units/DTU/software/homa (accessed on 10 April 2023)) ©The University of Oxford 2004–2021

3. The metabolic activity of osteocytes was assessed by measuring the subjects’ serum sclerostin concentration. Sclerostin was measured by an enzyme-linked immunosorbent assay (Soluble Sclerostin (SOST) (Human) ELISA Kit, AVISCERA BIOSCIENCE, INC, Santa Clara, CA, USA; catalog number SK00385-01)). The assay’s properties: sensitivity (detection limit)  ± 20 pg/mL, a broad detection range (125~4000 pg/mL), an intra-assay precision (Intra-CV) of 4–8% and an inter-assay precision (Inter-CV) of 6–10%.

Measurements were performed according to the manufacturer’s instructions.

The results were expressed in pg/mL between 125 and 1600.

The blood samples were collected by qualified staff of the hospital laboratory. Sclerostin concentration was assessed in the science laboratory at the Clinic of Endocrinology, Metabolic Diseases and Internal Diseases of Pomeranian Medical University in Szczecin, Poland.

4. All participants had their bone mineral density determined at lumbar spine (L1–L4) and for whole body by the dual-energy X-ray absorptiometry (DXA) technique (GE Lunar Prodigy Advance-Madison, WI, USA—z enCORE software version 8.8). Results were presented as BMD (g/cm^2^) and Z-score. According to the criteria, Z-score values >(−1.0) were stated as normal.

The participants were divided into two groups according to Z-score L1–L4. Group A (Z-score > −1.0), patients with normal bone mineral density, and Group B (Z-score < −1.0), with reduced BMD.

### 2.3. Statistical Analysis

The quantitative variables obtained in the statistical analysis were presented as mean, standard deviation (SD), median (M), and upper and lower quartiles. A cross-correlation analysis of bone mineralization values (BMDL1–L4, Z-score, BMC (Madison, WI, USA) using enCORE software (version 8.8)) and sclerostin concentration was carried out. When calculating the correlation of individual parameters, the number of N valid pairs was reported, due to missing individual data. Due to the diversification of sclerostin concentrations, patients were assigned to four groups, based on the median intermediate result (1163.5 pg/mL: below the test reference range (I ≤ 125 pg/mL), below the median value (II = 125–1163.5pg/mL), above the median value (III = 1163.5–16,000 pg/mL) and above the test reference range (IV = >16,000). The Shapiro–Wilk test was used to determine the normality of the distribution of continuous variables. The Brown–Forsythe test was used to determine variance homogeneity. Depending on the distribution of the variables, one-way ANOVA or Kruskal–Wallis rank ANOVA was used to compare the four groups. As a non-parametric post-hoc test, the Kruskal–Wallis test was used. Depending on the distribution of variables, the Student’s t-test or Mann–Whitney U test was used to compare the means of two groups. Quantitative variables were correlated using Pearson’s correlation for variables with a normal distribution and Spearman’s rank correlation for variables with a non-normal distribution. Correlation coefficient values of 0.01–0.19, 0.2–0.39, 0.4–0.59, 0.6–0.79, 0.8–0.99, and 1.0 were categorized as very weak, weak, moderate, strong, very strong, and perfect correlation, respectively. The interpretation was similar for negative values, but of a negative nature. Results with a *p*-value of 0.05 were considered statistically significant. Statistica 13.3 software was used for statistical analysis (TIBCO Software (version 13.3), PaloAlto, CA, USA). PQStat software (PQStat Software (version 1.84), Pozna, Poland) was used to create correlation charts.

## 3. Results

### 3.1. Characteristics of the Group

The characteristics of all the data collected for the calculations are shown in Table 1.

The characteristics of the patient groups divided according to the Z-score values of the BMD L1–L4 (group A: Z-score > −1.0 and group B: Z-score < −1.0) and their comparison are shown in Table 2.

Spearman’s rank correlation coefficients (R) between BMD L1–L4 bone mineral density and the parameters studied are shown in Table 3.

### 3.2. Hormone and Metabolic Parameters

In terms of the evaluated hormonal parameters, significant differences were found between the groups with normal and reduced bone mineral density in terms of concentrations of estradiol (*p* = 0.0189) and cortisol (*p* = 0.0070). There was no statistically significant correlation between the Z-score and testosterone and its derivatives (FAI/FT/BAT) or glucose, insulin, and the HOMA-IR index.

**Figure 1:**
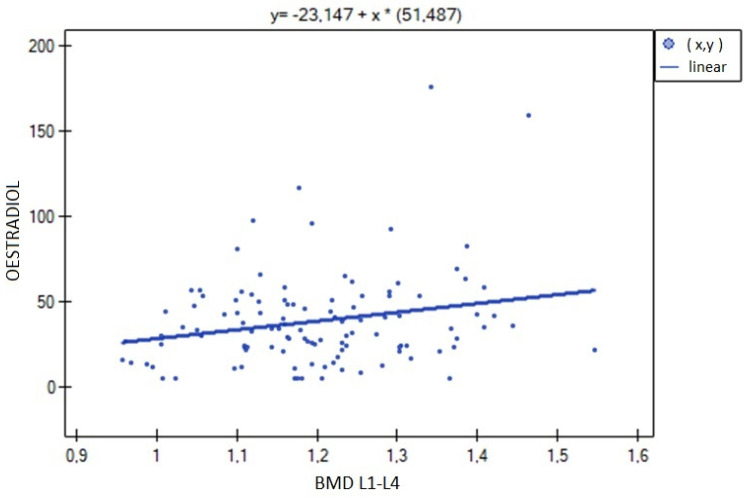
Relationship between L1–L4 BMD bone mineral density and estradiol concentration (R = 0.1239, *p* = 0.1949).

**Figure 2:**
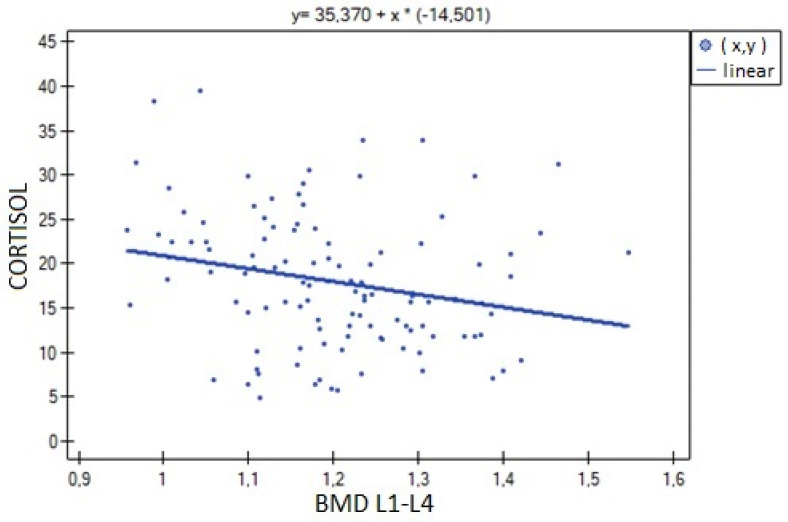
Relationship between L1–L4 BMD bone mineral density and cortisol concentration (R = −0.2633, *p* = 0.0052).

**Figure 3:**
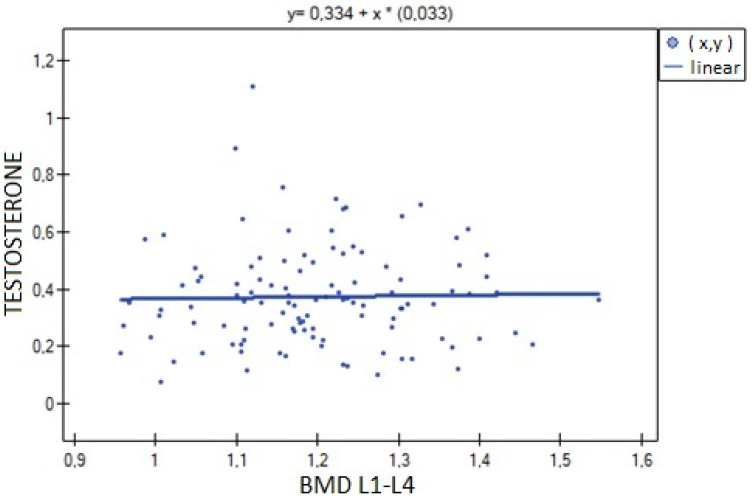
Relationship between BMD L1–L4 bone mineral density and testosterone concentration (R = 0.0600, *p* = 0.5312).

**Figure 4:**
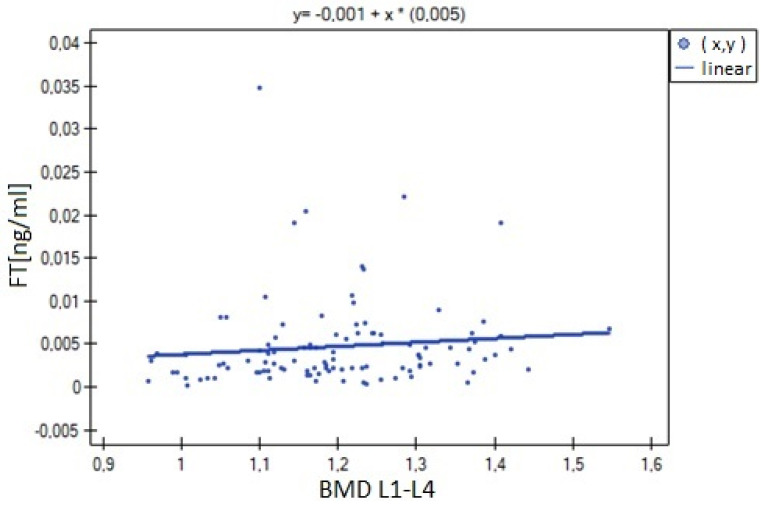
Relationship between BMD L1–L4 bone mineral density and free testosterone (FT) concentration (R = 0.2294, *p* = 0.0185).

**Figure 5:**
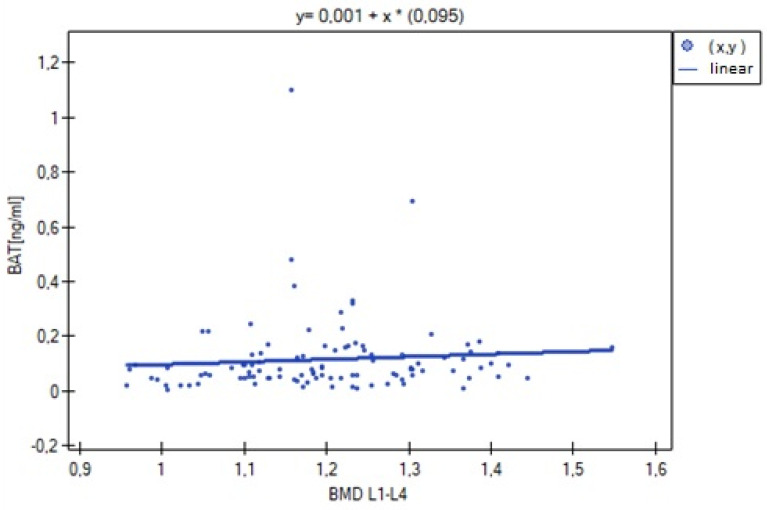
Correlation between L1–L4 BMD bone mineral density and bioavailable testosterone concentration (BAT) (R = 0.2002, *p* = 0.0405).

**Figure 6:**
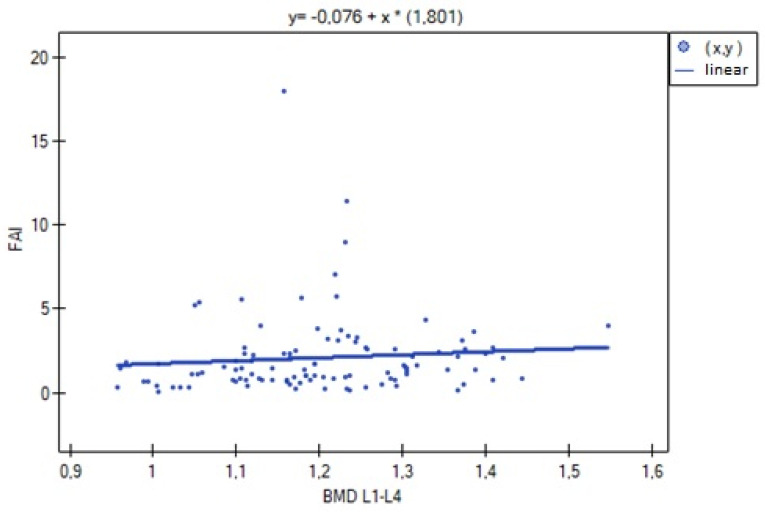
Relationship between bone mineral density BMD L1–L4 and free androgen index (FAI) (R = 0.2157, *p* = 0.0270).

The analysis of BMD L1–L4 showed a weak negative correlation with cortisol concentration (R= −0.2633, *p* = 0.0052), and no statistical significance was obtained for estradiol concentration (*p* = 0.194932). There was a weak positive correlation of BMD L1–L4 with free testosterone concentration (R = 0.2294, *p* = 0.0185), bioavailable testosterone (R = 0.2002, *p* = 0.0405) and the free androgen index (R = 0.2157, *p* = 0.0270). For testosterone concentration, the result was not statistically significant. The relationships described are shown in Figure 1, Figure 2, Figure 3, Figure 4, Figure 5 and Figure 6.

### 3.3. Sclerostin

The analysis of the data showed no differences of statistical significance in the parameters of bone mineralization (BMD total, BMD L1–L4, Z-score L1–L4, BMC) between the specified groups of sclerostin concentration (I, II, III, IV). The results also remained statistically insignificant when the sclerostin concentration was used as a quantitative variable.

A statistically significant correlation was found between sclerostin concentration and TSH (weak positive correlation, R = 0.2862, *p* = 0.0023). No statistically significant associations were found between sclerostin concentration and the other hormones and metabolic parameters studied.

## 4. Discussion

Osteoporosis is a significant global health care problem. Despite education of the public and the medical community, the disease is often diagnosed very late, usually after a complication such as an osteoporotic fracture. The latest epidemiological data was brought by the report ‘SCOPE 2021: a new scorecard for osteoporosis in Europe’. The document presents data on osteoporosis in the countries of the European Union, Switzerland and the United Kingdom. It was estimated that there were 25.5 million women and 6.5 million men with osteoporosis in 2019 in these countries. There were 4.3 million new osteoporotic fractures diagnosed, and the cost of treating them was approximately €57 billion [15]. Young women are a group of patients overlooked in research studies. It is surprising, because during this period it is possible to eliminate osteoporosis risk factors and achieve and maintain normal peak bone mass. The aim of this study was to fill this gap. Current trends in medicine place the highest emphasis on preventive actions. Therefore, increasing the knowledge of skeletal physiology, learning about modifiable risk factors for low peak bone mass and selecting individuals predisposed to osteoporosis will enable the implementation of early preventive measures. Even in non-menopausal women (aged 20–30 years), some hormonal markers (cortisol, FT, BAT, FAI) are already useful in assessing bone loss.

The present study showed a statistically significant association of bone mineral density with estradiol concentration and a negative relationship with cortisol concentration in the Z-score L1–L4. The results remain consistent with the current state of knowledge regarding bone physiology. Estrogen receptors are present in all bone cell types. In both sexes, estrogens play a key role in regulating bone metabolism. These conclusions were based on observations of women during the menopausal transition. The fundamental SWAN study (The Study of Women’s Health Across the Nation) brought a wealth of data on the physiology of the hypothalamic–pituitary–gonadal system and the effects of hormones on individual systems. The follow-up lasted 8 years, and 3302 women aged 42 and 52 years with at least one menstrual bleeding in the 3 months preceding the study were included. Urinary NTX concentration as an exponent of bone resorption, urinary creatinine concentration and E and FSH concentrations were assessed. As estrogen concentration decreased and FSH concentration increased, NTX concentration increased. During changes in the function of the gonadal axis in women, androgen secretion remains relatively constant, which excludes its influence on postmenopausal bone mass loss. Bone mineral density was assessed in 1902 women. In this study, subgroups were distinguished according to the frequency and regularity of menstrual bleeding: premenopause, early perimenopause, late perimenopause, and menopause. A significant decline in BMD already started during late perimenopause and continued until the early postmenopausal years. These women (over a period of about 5 years) lost about 7–10% of their initial bone mass of the lumbar spine. The loss was greater at lower body weights, which is naturally related to the stimulating role of mechanical loading [16]. The rate of bone density loss was inversely proportional to estradiol concentration and directly proportional to FSH concentration [17]; i.e., estrogens had a protective effect on BMD but not at each of the menopausal transition periods studied. Exogenous estrogens have a similar protective function [18].

Analysis of the importance of estrogens in the context of this study, confirms the necessity of their presence for normal mineralization. Assessing the relationship of androgens with bone mineralization appears problematic for several reasons. Testosterone in the bloodstream remains strongly associated with carrier proteins (SHBG, albumin) and assessment of the active hormone is uncertain. There are several indicators, also used in our study (FT, BAT, FAI), to calculate the bioavailable and reactive molecule. What is certain is that much of the action of testosterone on the skeleton depends on aromatization to estrogens. In the analysis of the relationship between androgens and bone mineralization in women, a prospective study by Slemend C. et al. is worth citing [19]. In the prospective evaluation, premenopausal (*n* = 94), perimenopausal (*n* = 28–62) and postmenopausal women (*n* = 73) were examined. In the participants, BMD of the femoral neck, lumbar spine (L2–L4) and radius bone was examined (at 0-, 6- and 12-month intervals) and hormonal parameters (estrogen, androgen, SHBG) were assessed. In the group of premenopausal women, it was shown that greater annual bone mass loss was associated with lower values of testosterone, free testosterone, and DHEA and DHEA-S concentrations. In a combined analysis of all study groups, lower T values were also found to be associated with reduced BMD [20].

The HERITAGE Family Study by He Z. et al. [21] involving a population from North America (USA, Canada) [21] and a large Swedish study involving 1200 men and an equal number of women aged 25–64 years by Ragnarsson O. et al. [22] showed a positive relationship of DHEA-S concentration with lean body mass. Referring to the above-described correlations of BMD with LBM, it can be speculated that this is another important mechanism for the effects of these hormones on the skeleton. The conclusions from the above work are in line with the results obtained in this study. Statistically significant relationships were found between L1–L4 BMD and the parameters FT, FAI and BAT, but not for total testosterone concentration. This confirms the greater effect of the free testosterone fraction on BMD described by Khosla S. et al. [23].

In the basal mechanism, insulin has an anabolic effect on bone. However, in a state of insulin resistance, it is present at concentrations above physiological limits, which appears to modify its effect. In experimental studies, a reduction in bone turnover associated with IR was found, which, despite its beneficial effect on aBMD, resulted in increased bone fragility. Human studies yield inconsistent conclusions, so the ultimate effect of insulin in the insulin-resistant state on the skeleton needs to be further investigated [13]. The study here did not show an association of bone mineralization with fasting glucose and insulin concentrations or the HOMA-IR insulin resistance index. Perhaps the lack of this relationship is due to the size of the study group.

Another significant relationship shown in this study concerns cortisol levels and BMD. It is generally known that glucocorticoid hormones (GCS) have a negative relationship with bone density, demonstrated mainly in studies on patients with exo- and endogenous Cushing’s syndrome. The most important appears to be the effect on bone-forming and bone-depleting cells. They cause a drastic loss of bone mass when cortisol is overproduced or when there is an excessive supply of its synthetic derivatives. However, their actions in the range of physiological concentrations are less clear [15,16].

There is an extremely limited number of studies on young adult women, especially healthy ones. One of the few studies on this topic, conducted by Bedford JL. et al. [17], analyses data on 140 women aged 19–35 years (mean 22.3 years), in good health, without menstrual disorders and non obese (BMI 18–30 kg/m^2^). Participants completed a stress assessment questionnaire (Perceived Stress Scale, PSS), gave daily urine collection samples for determination of free cortisol concentration (UFC), and bone density and mineral content were assessed by DXA. The analysis showed a negative correlation of bone parameters with the PSS score. Urinary free cortisol concentration was negatively associated with aBMD (total body and L1–L4) and BMC, also after correction for urine excretion volume. No association of UFC with PSS scores was demonstrated [17]. The conclusions obtained in the study described above are very important. It has been shown that cortisol concentrations, also within reference norms, have a negative effect on bone mineralization. The study population was healthy, so the possibility of interference in the results obtained can be excluded. The strength of the study is also enhanced by the method used to assess cortisol concentrations—24-h urine collection. The study here also found a negative effect of cortisol on lean body mass. Such a relationship is not surprising, given the known catabolic effect of cortisol on muscle. As with androgens, it can be assumed that this is another mechanism for the effect of this hormone on bone mineralization. The results of the study here: a significant negative correlation of cortisol concentration with L1–L4 BMD and L1–L4 Z-score, are in agreement with the literature examples cited above. Correct cortisol concentration is not the most significant variable determining bone density, but as one of many factors, it can significantly influence gained bone mass.

Doctors and nurses should be aware of these markers, even in non-menopausal women. Given that high cortisol levels may be associated with bone disorders, medical interventions should be established as early as possible, to prevent this problem. In non-menopausal women, high cortisol levels can be prevented by stress reduction and appropriate diet. Women who have shown lower levels of estrogens and androgens should be offered hormone supplementation and remain under constant medical supervision.

In the discussion on sclerostin, it is necessary to cite the paper by Ardawi MSM et al. from 2011 [18]. Compared to the studies cited above, the researchers obtained different, more expected, results. The study group consisted of 1803 healthy Saudi women aged 20 to 79 years (premenopausal *n* = 1235 and postmenopausal *n* = 568). There was a linear increase in sclerostin levels proportional to the age of the subjects. Before menopause, there were negative correlations of sclerostin with bone density (BMD L1–L4, femoral neck BMD) and estradiol levels (testosterone levels were not included in the study program). The authors conclude that increasing sclerostin concentrations may be both a cause and a consequence of bone mass loss. In our study, very low concentrations of sclerostin, below the detection threshold, were observed. Theoretically, this could be due to physiologically low concentrations of this parameter in young healthy individuals, when their bone mass reaches its maximum, or it could indicate low sensitivity of the test used. For this reason, both statistical analysis and final conclusions were handicapped. The reliability of the test could be confirmed by performing the determination on a larger group of patients.

The strong negative association of estradiol and sclerostin concentrations is in line with knowledge of the effects of female sex hormones on the skeleton [18]. Low concentrations of estrogens predispose the patient to bone mass loss, so an association with higher concentrations of sclerostin seems obvious. In our study, we did not find a similar relationship. Similarly, an association of SOST with parameters expressing bone mineralization was not proven. The reason for this may be due to the much smaller study group and the distribution of data obtained in the sclerostin measurement.

## 5. Conclusions

It has been shown that test hormone concentrations, even within the range of reference norms, can affect bone mineralization. We suggest observing the follow-up of menstrual cycles, as well as analyzing the results of test patients in an annual examination system. However, each clinical case should be considered individually. Sclerostin testing is not yet useful for the clinical assessment of bone mineralization in young adult women.

## Figures and Tables

**Table 1:** Statistical characteristics of the studied parameters of female participants.

Variable	*n*	Average	Median	Q1	Q4	SD *
Age [years]	111	23.80	24.00	23.00	25.00	1.793
Body weight [kg]	111	63.48	61.00	56.00	68.00	11.854
Height [cm]	111	168.21	168.00	164.00	173.00	5.967
BMI [kg/m^2^]	111	22.39	21.00	19.50	23.50	4.223
BMD Total [g/cm^2^]	111	1.14	1.13	1.06	1.21	0.096
BMD L_1_–L_4_ [g/cm^2^]	111	1.20	1.19	1.11	1.29	0.122
BMD L_1_–L_4 z-score_	111	0.22	0.10	−0.50	0.80	1.001
BMC [g]	111	2390.55	2407.00	2220.00	2624.00	351.032
MS-R [kg]	111	27.96	28.30	24.70	31.30	4.395
MS-L [kg]	111	26.38	26.30	23.10	30.40	4.781
FLR	111	0.50	0.45	0.38	0.58	0.180
A/G	111	0.79	0.79	0.63	0.93	0.202
VF [g]	111	273.44	161.00	60.00	309.00	355.636
Glucose [mg/dL]	109	88.44	89.00	83.00	93.30	7.616
Insulin [µIU/mL]	106	10.43	9.36	7.10	12.90	5.267
HOMA2 IR	106	1.34	1.20	0.91	1.64	0.666
Estradiol [pg/mL]	111	38.55	33.21	22.10	50.60	27.827
Testosterone [ng/mL]	111	0.37	0.35	0.25	0.47	0.174
FT [ng/mL]	105	0.00	0.00	0.00	0.01	0.005
BAT [ng/mL]	105	0.12	0.08	0.05	0.14	0.140
FAI	105	2.08	1.40	0.76	2.56	2.415
Cortisol [ng/mL]	111	17.99	17.60	12.00	22.70	7.572
TSH [μIU/mL]	111	2.11	1.76	1.19	2.80	1.411
Sclerostin [pg/mL]	111	2136.37	125.00	125.00	263.00	4989.928

MS–R—right-hand muscle strength, MS–L—left-hand muscle strength, SD—standard deviation, HOMA 2 IR—insulin resistance index, FT—free testosterone, BAT—bioavailable testosterone, FAI—free androgen index, FLR—fat–lean ratio, A/G—android/gynoid ratio, VF—visceral fat mass, BMD Total—total bone mineral density, BMD L1–L4—bone mineral density of the L1–L4 segment, BMD L1–L4 z-score—bone mineral density Z-score (standard deviation from mean value), BMC—bone mineral content, * *n*—number of variables, Q1—lower quartile, Q4—upper quartile, SD—standard deviation.

**Table 2:** Statistical characteristics and comparison of study parameters of female participants in groups A (with normal bone mineral density) and B (with reduced bone mineral density).

Variable	Group A			Group B			Value *p*
	*n*	Mean	Q1–Q4	*n*	Mean	Q1–Q4	
		(SD/Median)			(SD/Median)		
FM [kg]	95	20.20	14.34–22.93	16	23.89	15.53–32.19	>0.1
		(8.76/18.17)			(11.89/17.93)		
LBM [kg]	95	41.03	38.04–43.61	16	40.58	36.09–45.78	>0.1
		(4.40/41.29)			(4.78/39.43)		
Glucose [mg/dl]	93	88.26	83.50–93.20	16	89.48	82.30–94.90	>0.1
		(7.68/89.00)			(7.37/89.10)		
Insulin [µIU/mL]	90	10.10	6.80–12.60	16	12.30	8.26–14.10	>0.1
		(4.87/9.15)			(7.03/12.15)		
HOMA2 IR	90	1.30	0.88–1.59	16	1.59	1.06–1.85	>0.1
		(0.62/1.18)			(0.87/1.56)		
Estradiol [pg/mL]	95	40.91	23.20–53.40	16	24.54	13.66–33.21	0.0189
		(29.00/35.30)			(12.75/25.80)		
Testosterone [ng/mL]	95	0.38	0.25–0.48	16	0.37	0.25–0.47	>0.1
		(0.18/0.36)			(0.18/0.34)		
FT [ng/mL]	90	0.00	0.00–0.01	15	0.00	0.00–0.01	>0.1
		(0.18/0.36)			(0.01/0.00)		
BAT [ng/mL]	90	0.12	0.05–0.14	15	0.11	0.02–0.22	>0.1
		(0.14/0.08)			(0.13/0.05)		
FAI	90	1.95	0.81–2.56	15	2.87	0.37–5.23	>0.1
		(1.83/1.42)			(4.61/0.79)		
Sclerostin [pg/mL]	95	1950.59	125.00–244.00	16	3239.45	125.00–1228.12	>0.1
		(4740.95/125.0)			(6342.35/125.00)		
Cortisol [ng/mL]	95	17.24	11.70–21.63	16	22.49	18.65–25.17	0.0070
		(7.42/16.00)			(7.12/22.89)		

HOMA 2 IR—insulin resistance index, FT—free testosterone, BAT—bioavailable testosterone, FAI—free androgen index, FM—fat mass, LBM–lean body mass, *n*—number of variables, SD—standard deviation, Q1—lower quartile, Q4—upper quartile.

**Table 3:** Relationship between BMD L1–L4 bone mineral density and the parameters studied.

Variable	*n*	R	*p*
FLR	111	0.2042	0.0315
VF [g]	111	0.1586	0.0963
Glucose [mg/dL]	109	0.1120	0.2460
Insulin [µIU/mL]	106	−0.0497	0.6122
HOMA2 IR	106	−0.0422	0.6668
Estradiol [pg/mL]	111	0.1239	0.1949
Testosterone [ng/mL]	111	0.0600	0.5312
FT [ng/mL]	105	0.2294	0.0185
BAT [ng/mL]	105	0.2002	0.0405
FAI	105	0.2157	0.0270
Cortisol [ng/mL]	111	−0.2633	0.0052
Sclerostin [pg/mL]	111	0.0204	0.8314

HOMA 2 IR—insulin resistance index, FT—free testosterone, BAT—bioavailable testosterone, FAI—free androgen index, FLR—fat–lean ratio, VF—visceral fat mass, *n*—number of variables, R—Spearman’s/ Pearson’s rank correlation coefficients, *p*—*p*-value.

## Data Availability

Data are available on special request after contacting the author (M.P.-W.).
